# Reconsidering the Role of Melatonin in Rheumatoid Arthritis

**DOI:** 10.3390/ijms21082877

**Published:** 2020-04-20

**Authors:** Iona J. MacDonald, Chien-Chung Huang, Shan-Chi Liu, Chih-Hsin Tang

**Affiliations:** 1 School of Medicine, China Medical University, Taichung 40402, Taiwan; ionamac@gmail.com (I.J.M.); u104054003@cmu.edu.tw (C.-C.H.); 2 Division of Immunology and Rheumatology, Department of Internal Medicine, China Medical University Hospital, Taichung 40447, Taiwan; 3 Department of Medical Education and Research, China Medical University Beigang Hospital, Yunlin 65152, Taiwan; sdsaw.tw@yahoo.com.tw; 4 Graduate Institute of Biomedical Sciences, China Medical University, Taichung 40402, Taiwan; 5 Chinese Medicine Research Center, China Medical University, Taichung 40402, Taiwan; 6 Department of Biotechnology, College of Health Science, Asia University, Taichung 41354, Taiwan

**Keywords:** melatonin, rheumatoid arthritis

## Abstract

Rheumatoid arthritis (RA) is an inflammatory joint disorder characterized by synovial proliferation and inflammation, with eventual joint destruction if inadequately treated. Modern therapies approved for RA target the proinflammatory cytokines or Janus kinases that mediate the initiation and progression of the disease. However, these agents fail to benefit all patients with RA, and many lose therapeutic responsiveness over time. More effective or adjuvant treatments are needed. Melatonin has shown beneficial activity in several animal models and clinical trials of inflammatory autoimmune diseases, but the role of melatonin is controversial in RA. Some research suggests that melatonin enhances proinflammatory activities and thus promotes disease activity in RA, while other work has documented substantial anti-inflammatory and immunoregulatory properties of melatonin in preclinical models of arthritis. In addition, disturbance of the circadian rhythm is associated with RA development and melatonin has been found to affect clock gene expression in joints of RA. This review summarizes current understanding about the immunopathogenic characteristics of melatonin in RA disease. Comprehensive consideration is required by clinical rheumatologists to balance the contradictory effects.

## 1. Introduction

Rheumatoid arthritis (RA) is an autoimmune disease that is characterized by synovial proliferation and inflammatory responses, the presence of autoantibodies including rheumatoid factor and anti-citrullinated protein antibodies (ACPA) in sera, cartilage, and bone erosion with deformity, and co-occurring health conditions such as cardiovascular disease events, pulmonary, psychological, and metabolic bone disorders [1]. Proinflammatory cytokines that mediate the progression of RA disease include tumor necrosis factor alpha (TNF-α), interleukin 1 beta (IL-1β), and IL-6. Current international guidelines for patients with early RA recommend starting disease-modifying antirheumatic drugs (DMARDs) as soon as possible, with methotrexate being the preferred choice [2]. Methotrexate is usually supplemented with short-term, low-dose oral or intra-articular glucocorticoids (GCs) for fast relief of pain and swelling and for arresting the inflammatory process. GCs must be carefully managed to prevent their inappropriate use and tapered as soon as possible to avoid long-term adverse effects [2]. The highly efficacious biologic DMARDs targeting the proinflammatory cytokines and Janus kinase inhibitors are intended for patients with persistently active disease after initial methotrexate failure and, in some cases, another conventional DMARD [2]. However, although these novel medications help to control RA disease activity, they are not universally effective in all RA patients [3,4] and many will lose therapeutic responsiveness after a period of time [5]. More potent or complementary treatments are needed.

The anti-inflammatory and antioxidant effects of melatonin have proven beneficial in several inflammatory autoimmune diseases [6]. Melatonin is also capable of regulating responses of T cell subsets, such as CD4^+^ T helper (Th)1, Th17, and regulatory T cells (Tregs) [7]. However, numerous studies indicate that melatonin, instead of being beneficial, could exacerbate RA disease-related activities. Nevertheless, in recent years, some conflicting results show that melatonin is capable of alleviating RA through anti-inflammatory and immunoregulatory mechanisms. The aim of this review is to elucidate the complex reactions of melatonin in RA and determine whether melatonin could serve as a potential therapeutic agent.

## 2. Mechanisms of Melatonin

Endocrine circadian rhythms are regulated by the endogenous hormone melatonin (*N*-acetyl-5-methoxytryptamine), which activates specific high-affinity melatonin receptors expressed on several different types of cells, including immunocompetent cells [8]. The pineal gland is the primary source of melatonin, which is also synthesized by tissues and organs of multicellular organisms including the retina, gastrointestinal tract, skin, and leukoctyes (in peripheral blood and in bone marrow) [9]. In the skin, melatonin can regulate cutaneous pigmentation and perform photoprotective and anticancer activities [10,11]. *N*-acetylserotonin, a precursor to melatonin, enters the systemic circulation from different peripheral organs for transformation to melatonin [12]. Furthermore, the effects of melatonin can be mediated by its metabolites because of the rapid metabolism of melatonin at the peripheral sites [13,14]. As the production of melatonin by the non-endocrine organs responds to signals other than circadian cycles, the endocrine, autocrine, and paracrine effects of melatonin mean that this substance is extensively involved in the regulation of the human immune system [9].

The biological effects of melatonin involve three pathways: (i) G-protein-coupled membrane receptor signaling; (ii) nuclear signaling; and (iii) receptor-independent signaling that accounts for radical scavenging activities of melatonin [7,15]. The binding of melatonin to the G protein-coupled melatonin receptors (MT_1_ and MT_2_) on the plasma membrane of the target cells enables melatonin to stimulate signaling pathways and reduce cell proliferation [16]. MT_1_ receptors are expressed throughout the body but mainly in the central nervous system, in the thymus and the spleen, B cells, CD4, and CD8 cells [7]. Neuroanatomical mapping of melatonin receptors has revealed marked differences in distribution patterns of MT_1_ and MT_2_ proteins in the adult rat brain, with for instance MT_2_ receptors identified in the reticular thalamic nucleus, mediating neuronal firing and burst activity related to nonrapid eye movement (NREM) sleep, whereas no MT_1_ receptors are found in this area, confirming the highly specific functioning of melatonin receptors in sleep neurophysiology [17]. MT_1_ and MT_2_ affect gene transcription activities through extracellular signal-regulated kinase (ERK) pathways and CREP phosphorylation [7]. Melatonin receptors have also been identified in RA synovial macrophages [18]. A positive correlation has been observed between the polymorphism of the melatonin receptor type 1B (*MTNR1B*) and levels of rheumatoid factor in Korean patients with RA [19]. Other researchers have reported significantly lower levels of MT_1_ expression in RA synovial tissue compared with normal healthy tissue, and the finding that siRNAs against MT_1_ reverse melatonin-mediated inhibition of TNF-α and IL-1β production, confirming that melatonin suppresses TNF-α and IL-1β via the MT_1_ receptor [20]. Thus, the activity of membrane melatonin receptors and their specific agonists is implicated in circadian rhythmicity [7]. Retinoic acid-related orphan receptor alpha (RORα) is an important member of the ROR subfamily of nuclear receptors, which mediate several physiological functions, such as metabolic, immunologic, and circadian actions [21]. The identified endogenous ligands for RORα consist of sterols and their derivatives [22]. It appears that RORα mediates the indirect effects of melatonin in the periphery, such as immunomodulation, cellular growth, and bone differentiation [23]. Moreover, while RORα is not a receptor for melatonin or its metabolites, the constitutive activity of RORα may be modulated by membrane melatonin receptors [21,23].

Melatonin also displays antioxidant and anti-inflammatory activity, depending on the cellular state [24,25]. Evidence suggests that melatonin serves as a link between circadian rhythms and joint diseases, including RA and osteoarthritis (OA) [26,27]. For instance, oxidative stress induced by RA is reduced by melatonin and/or its metabolites, which not only neutralize the reactive oxygen (ROS) and reactive nitrogen species (RNS), but also upregulate levels of glutathione and antioxidant enzyme expression and activity [28,29]. In RA and OA, melatonin and its metabolites modulate several molecular signaling pathways including those governing inflammation, proliferation, and apoptosis [28,29].

## 3. A Role for Melatonin in Rheumatoid Arthritis Therapy?

In investigations involving melatonin in animal models of inflammatory autoimmune diseases (multiple sclerosis, systemic lupus erythematosus, inflammatory bowel disease, and type 1 diabetes), melatonin has demonstrated beneficial effects in these diseases, including prophylactic and therapeutic effects in rats with adjuvant-induced arthritis (AA), in which melatonin dose-dependently repressed the inflammatory response and enhanced proliferation of thymocytes and secretion of IL-2 [30]. In addition, melatonin decreased the elevated level of cyclic 3′,5′-AMP (cAMP) induced by forskolin. The drop in thymocyte proliferation induced by injection of Freund’s complete adjuvant was highly correlated with a decrease in the levels of Met-enkephalin (Met-Enk) in the thymocytes, which were strikingly augmented by melatonin; this effect was blocked by the Ca^2+^ channel antagonist, nifedipine. The anti-inflammatory and immunoregulatory actions of melatonin involved a G protein-adenyl cyclase-cAMP transmembrane signal and Met-Enk release by thymocytes [30].

### 3.1. Modulation of the Circadian Clock by Melatonin in RA

Importantly, circadian rhythms exist in almost all cells of the body, and are regulated by circadian clock gene expression [7,15]. Any disruption in these circadian clocks is associated with the onset of inflammatory-related disease states and joint diseases, including RA [7,15]. Patients with RA exhibit abnormal clock gene expression, with disturbances in the hypothalamic-pituitary-adrenal axis influencing changes in circadian rhythms of circulating serum levels of melatonin, IL-6, cortisol and in chronic fatigue [15]. Melatonin exerts its effects in RA by modulating clock gene expression, including the *Cry1* gene [7,15]. By attenuating the expression of the *Cry1* gene, melatonin upregulates levels of cAMP production and increases activation of protein kinase A (PKA) and nuclear factor kappa B (NF-κB), which increases CIA severity in rats [31,32]. As detailed earlier, the diurnal secretion of melatonin is also closely related to the production of IL-12 and NO among RA synovial macrophages and human monocytic myeloid THP-1 cells [33].

A positive correlation has been reported between elevated early morning serum melatonin concentrations and disease activity scores as well as erythrocyte sedimentation rate (ESR) levels in patients with juvenile rheumatoid arthritis, although higher melatonin concentrations did not correlate with disease severity [34], echoing the findings of Forrest and colleagues reported earlier, who noted that elevated ESR and neopterin concentrations following melatonin treatment did not worsen the severity of RA disease [35]. El-Awady and colleagues suggested that melatonin may promote the activity of RA disease, rather than its severity [34]. However, as reported above, Akfhamizadeh and colleagues found no link between elevated morning serum melatonin concentrations and RA disease activity or other disease characteristics, despite also observing significantly higher melatonin values in newly diagnosed RA patients compared with those who had established RA disease [36].

There also appears to be a relationship between melatonin and the *Bmal1* and *ROR* clock genes [15]. It is speculated that high melatonin concentrations in RA patients may modulate *ROR* activation [15]. *ROR* acts as a negative regulator of inflammation via the NF-κB signaling pathway and is essential in the activity of both melatonin and the clock gene *Bmal1*, which helps to maintain 24-h rhythms and regulate immune responses [15,37]. Moreover, ROR proteins bind into the promoter region and drive *Bmal1* gene expression [38]. This activity at the binding site is inhibited by reverse-eritroblastosis viruses (REV-ERBs), which may contribute to *Bmal1* suppression and exacerbation of RA [15].

### 3.2. Adverse Effects of Melatonin in RA

Evidence suggests that melatonin is not beneficial in RA. For instance, the development of collagen-induced arthritis (CIA) in DBA/1 mice is exacerbated by constant darkness [39] and by daily exogenous administration of melatonin 1 mg/kg [40]. Hansson and colleagues then investigated the effects of surgical pinealectomy in DBA/1 and NFR/N mice with collagen-induced arthritis (CIA) [41]. Serum melatonin levels were reduced in the pinealectomized mice to around 30% of levels in normal or sham-operated controls [41]. In both mouse strains, pinealectomy was associated with a delay in onset of arthritic disease, less severe arthritis (lower clinical scores), and lower serum anti-CII levels compared with sham-operated animals [41]. The researchers interpreted these findings as showing that high physiological levels of melatonin stimulate the immune system and worsen CIA, while inhibiting the release of melatonin is beneficial [41]. Their speculation was supported by observations from mice subjected to 30 days of Bacillus Calmette-Guérin (BCG) inoculations into the left hind paw, inducing chronic granulomatous inflammation [42]. Higher vascular permeability was seen around the granulomatous lesions at midnight than at midday; this rhythmic variation was eliminated by pinealectomy and restored by nocturnal replacement of melatonin [42].

This ability of melatonin to modulate immune response was further illustrated by experiments in which the production of IL-12 and nitric oxide (NO) was significantly increased in the media of melatonin-stimulated RA synovial macrophages and cultured THP-1 cells compared with RPMI-treated synovial macrophage controls [33]. Unexpectedly, the opposite effects in IL-12 and NO levels were seen when RA synovial macrophages were pretreated with lipopolysaccharide (LPS) prior to melatonin, as compared with synovial macrophages treated with LPS alone [33]. This study explained the possible mechanism of joint morning stiffness in relation to diurnal rhythmicity of neuroendocrine pathways [33].

These conclusions are supported by later evidence from in vitro and in vivo studies, as well as clinical investigations, showing how melatonin stimulates the production of NO, T helper type 1 (Th1)-type and other inflammatory cytokines besides IL-12 (IL-1, IL-2, IL-4, IL-5, IL-6, TNF-α, granulocyte-macrophage colony-stimulating factor [GM-CSF], and transforming growth factor [TGF]-β, interferon [IFN]-γ), and enhances both cell-mediated and humoral responses [43,44,45]. In the early morning, patients with RA exhibit high serum levels of proinflammatory cytokines, especially TNF-α and IL-6, when melatonin serum concentrations are also higher [6,43]. The effects of these circadian rhythms are thought to promote the joint pain and morning stiffness that characterizes RA [6]. Animal studies have shown that melatonin treatment (10 mg/kg) dysregulates circadian clock genes, which may promote the progression of RA [31]. Intriguingly, a dual effect of melatonin as a proinflammatory agent and antioxidant has been observed in CIA rats [32]. In that study, a lower dosage of melatonin (30 µg/kg) increased anti-collagen antibodies, IL-1β, and IL-6 levels in the serum and joints of arthritic rats, worsening the severity of joint damage, while simultaneously lowering oxidative markers nitrite/nitrate and lipid peroxidation in serum, but not in joints [32].

### 3.3. Neutral or Beneficial Effects of Melatonin in RA

Notably, a cross-sectional study from Iran has reported finding significantly higher morning serum levels of melatonin in patients with RA compared with healthy controls, but no correlation between melatonin and RA disease activity score or other disease characteristics, including age, disease duration, medications, gender, or season of sampling [36]. The study also reported finding higher serum melatonin values in newly diagnosed patients compared with patients with established RA, which needs further investigation [36].

Matrix metalloproteinases (MMPs) are a family of endopeptidases primarily responsible for catalyzing the degradation of the extracellular matrix (ECM) [46]. MMPs play important roles in RA. Elevated levels of circulating MMP-3, MMP-8 and MMP-9 are associated with disease progression in RA [47]. In particular, MMP-2 and MMP-9 are expressed in synoviocytes, CD34^+^ endothelial cells, monocytes and macrophages of rheumatoid synovium, indicating that both molecules are critical to pannus formation and invasion in RA progression [47]. Interestingly, melatonin reportedly directly inhibits secreted MMP-9 by binding to the active site and significantly reducing the catalytic activity of MMP-9 in both in vitro and cultured cells, in a dose- and time-dependent manner [46]. Thus, melatonin could have an important role in the prevention of joint destruction in RA.

Research demonstrating that melatonin dose-dependently inhibits the proliferation of RA fibroblast-like synoviocytes (FLS) through the activation of the ERK/P21/P27 pathway suggests that inhibiting the invasion of RA FLS through cartilage and into bone may have important implications in the treatment of RA [48]. Blocking NF-κB signaling appears to be the way in which melatonin protects cells from oxidative stress [28] and largely explains how melatonin suppresses proinflammatory cytokines such as IL-1β and TNF-α [20]. Other pathways and molecules associated with inflammation that are modulated by melatonin include the mitogen-activated protein kinase (MAPK) and nuclear erythroid 2-related factor 2 (Nrf2) pathways, as well as Toll-like receptors [15].

In a clinical trial involving RA patients, six months of melatonin treatment (10 mg/day) was associated with a general decrease from baseline in concentrations of peroxidation markers [35]. Conversely, ESR and neopterin concentrations were increased from baseline with melatonin and significantly higher at six months than concentrations in the placebo-treated cohort, which experienced a significant downward trend in these inflammatory indicators during the trial [35]. Paradoxically, neither the elevations in ESR and neopterin concentrations nor the decrease in tissue peroxidation associated with melatonin translated into significant differences from the placebo group in terms of patients’ symptoms, or in the concentrations of proinflammatory cytokines (TNF-α, IL-1β, and IL-6) [35]. The study researchers concluded that melatonin does not appear to be beneficial in RA [35].

The findings of Forrest et al. are discussed by Maestroni et al., who argue that because melatonin enhances the production of Th1-type and inflammatory cytokines in RA, upregulates cell-mediated and humoral responses, and also exacerbates CIA in mice, melatonin likely promotes RA disease and is inappropriate for therapeutic use [49]. Maestroni et al. also emphasized the high blood melatonin concentrations (280 pg/mL) observed 12 h after dosing in the RA cohort [49], in relation to the notion that higher blood melatonin concentrations, especially in the early morning, may be responsible for morning stiffness and joint swelling experienced by patients with arthritis [6,43]. Maestroni et al. conjecture that autoreactive T cells in RA patients synthesize and release melatonin, thereby worsening the disease process [49].

Nevertheless, Korkmaz [50] has defended Forrest et al., pointing out that melatonin has shown strong anti-inflammatory activity in studies using known inflammatory agents, such as zymosan [51], lipopolysaccharide [52,53], and carrageenan [54]. Korkmaz speculated that the high blood melatonin concentrations in the RA cohort may have been a compensatory response to RA inflammation and were comparable to the high levels of melatonin in cerebrospinal fluid and its metabolites in meningitis populations and in pediatric patients with epilepsy [50]. Korkmaz maintained that melatonin is an appropriate adjunctive therapy for RA [50].

**Figure 1:**
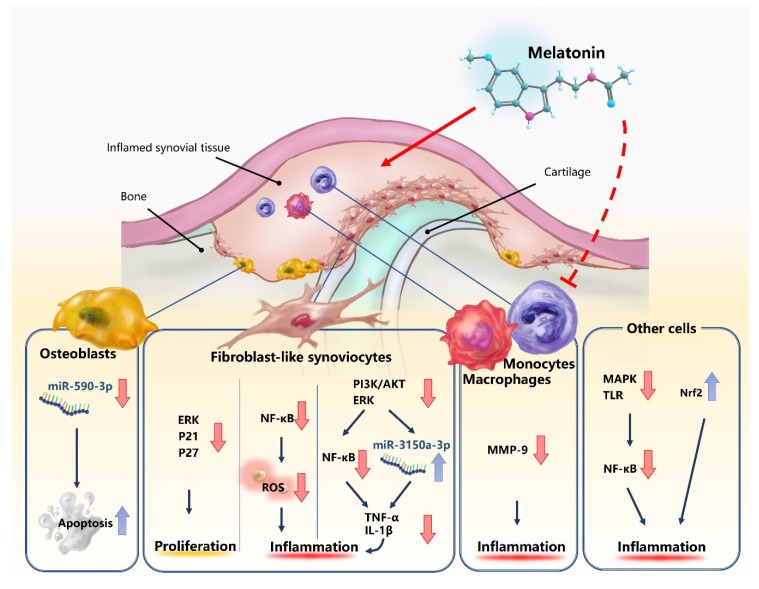
Neutral or beneficial effects of melatonin in rheumatoid arthritis (RA). The dotted line represents the uncertainty over the effects of melatonin upon macrophages and monocytes.

Melatonin appears to play an important role in microRNA (miRNA) expression in RA. miRNAs are small, non-coding RNAs that post-transcriptionally mediate protein expression by targeting protein-coding genes implicated in cancer cell proliferation, differentiation, apoptosis, and migration [55]. A recent study found that melatonin appears to inhibit miR-590-3p expression and induce apoptosis in human osteoblasts [55]. In another study, melatonin treatment effectively downregulated TNF-α and IL-1β production in human RA synovial fibroblasts (the MH7A cell line) by suppressing PI3K/AKT, ERK, and NF-κB signaling and upregulating miR-3150a-3p expression [20]. Those investigations confirmed that the MT_1_ receptor mediates the anti-inflammatory effects of melatonin and that melatonin not only inhibits inflammatory cytokine release in mice with CIA-induced arthritis, but also attenuates CIA-induced cartilage degradation and bone erosion [20]. This evidence suggests that melatonin targets miRNAs, which could be explored in clinical trials examining the efficacy of melatonin in the treatment of RA. The following figure (Figure 1) and table (Table 1) illustrate the processes through which melatonin exerts its therapeutic effects.

## 4. Summary

Various research suggests that melatonin has disease-promoting effects in RA and that it could increase the severity of RA, in contradiction to the beneficial effects of melatonin in other autoimmune inflammatory diseases. However, in the past decade, some studies have demonstrated that melatonin can alleviate RA through the inhibition of RA synovial fibroblast proliferation, TNF-α and IL-1β expression, as well as MMP-9 activity. The anti-inflammatory character of melatonin in RA is associated with regulation of microRNAs (such as miR-3150a-3p). More investigations are therefore warranted to explore the possible double-edged effects of melatonin in RA. The use of melatonin in patients with RA needs thorough consideration by clinical physicians.

## Figures and Tables

**Table 1:** Summary of the adverse, neutral, and beneficial effects of melatonin in rheumatoid arthritis.

Adverse/Neutral/Beneficial	Sample/Animal Model/Cell Line	Melatonin Level	Effects		References
Adverse	CIA mice	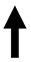	RA	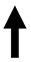	[40]
Adverse	CIA mice (pinealectomy)	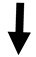	Serum anti-CII RA	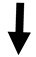	[41]
Adverse	RA synovial macrophages/myeloid monocytic cells (THP-1)	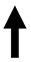	IL-12 NO	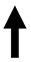	[33]
Adverse	CIA rats	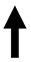	Anti-collagen antibodies IL-1β and IL-6	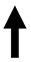	[32]
Neutral		Treatment with melatonin	Erythrocyte sedimentation rate (ESR) and neopterin	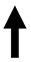	[35]
Neutral	RA patients’ plasma levels	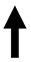			[36]
Beneficial		Treatment with melatonin	MMP-9	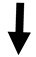	[46]
Beneficial	Fibroblast-like synoviocytes (FLS)	Treatment with melatonin	P21/P27 FLS proliferation	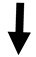 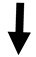	[48]
Beneficial	Synovial fibroblasts	Treatment with melatonin	miR-3150a-3p IL-1β and TNF-α	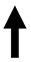 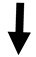	[20]
Beneficial		Treatment with melatonin	TLR/MAPK Nrf2	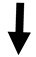 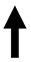	[15]
Beneficial		Treatment with melatonin	ROS and RNS glutathione and antioxidant enzymes	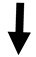 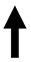	[28]
Beneficial	Osteoblast cells	Treatment with melatonin	miR-590-3p Apoptosis	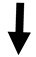 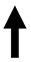	[55]

## Abbreviations

AA	Adjuvant-induced arthritis
ACPA	Anti-citrullinated protein antibodies
BCG	Bacillus Calmette-Guérin
Ca^2+^	Calcium
cAMP	Cyclic 3′,5′-AMP
CIA	Collagen-induced arthritis
DMARD	Disease-modifying antirheumaticdrug
ECM	Extracellular matrix
ERK	Extracellular signal-regulated kinase
ESR	Erythrocyte sedimentation rate
FLS	Fibroblast-like synoviocytes
GM-CSF	Granulocyte-macrophage colony-stimulating factor
IFN	Interferon
IL	Interleukin
Met-Enk	Met-enkephalin
MMP	Matrix metalloproteinase
MT_1/2_	High-affinity G protein-coupled melatonin receptors
NF-κB	Nuclear factor kappa B
NO	Nitric oxide
Nrf2	Nuclear erythroid 2-related factor 2
OA	Osteoarthritis
PI3K	Phosphatidylinositol 3-kinase
RA	Rheumatoid arthritis
ROS	Reactive oxygen species
TGF	Transforming growth factor
Th1	Human T helper type 1
TNF	Tumor necrosis factor
